# The interaction between NOLC1 and IAV NS1 protein promotes host cell apoptosis and reduces virus replication

**DOI:** 10.18632/oncotarget.21785

**Published:** 2017-10-10

**Authors:** Chunyu Zhu, Fangliang Zheng, Junfeng Zhu, Meichen Liu, Na Liu, Xue Li, Li Zhang, Zaidong Deng, Qi Zhao, Hongsheng Liu

**Affiliations:** ^1^ Key Laboratory of Animal Resource and Epidemic Disease Prevention, School of Life Science, Liaoning University, Shenyang 110036, China; ^2^ Research Center for Computer Simulating and Information Processing of Bio-Macromolecules of Liaoning, Shenyang 110036, China; ^3^ School of Mathematics, Liaoning University, Shenyang 110036, China

**Keywords:** IAV, NS1, NOLC1, protein-protein interaction, virus replication

## Abstract

NS1 of the influenza virus plays an important role in the infection ability of the influenza virus. Our previous research found that NS1 protein interacts with the NOLC1 protein of host cells, however, the function of the interaction is unknown. In the present study, the role of the interaction between the two proteins in infection was further studied. Several analyses, including the use of a pull-down assay, Co-IP, western blot analysis, overexpression, RNAi, flow cytometry, etc., were used to demonstrate that the NS1 protein of H3N2 influenza virus interacts with host protein NOLC1 and reduces the quantity of NOLC1. The interaction also promotes apoptosis in A549 host cells, while the suppression of NOLC1 protein reduces the proliferation of the H3N2 virus. Based on these data, it was concluded that during the process of infection, NS1 protein interacts with NOLC1 protein, reducing the level of NOLC1, and that the interaction between the two proteins promotes apoptosis of host cells, thus reducing the proliferation of the virus. These findings provide new information on the biological function of the interaction between NS1 and NOLC1.

## INTRODUCTION

Influenza A virus (IAV) is a human respiratory pathogen that causes seasonal epidemics and occasional global pandemics with devastating levels of morbidity and mortality. The non-structural protein 1 (NS1) of influenza A virus is a non-structural protein composed of 202-237 amino acids with a molecular mass of ∼28kD [1, 2], it is coded by Segment 8 of the influenza virus genome [3]. Being a protein with pleiotropic functions, NS1 has a variety of cellular interaction partners [4]. It has two functional domains: an amino-terminal RNA binding domain and a carboxyl-terminal effector domain [5, 6], which allow the NS1 protein to bind RNA [7], DNA [8] and/or interact with various multifunctional host proteins that are associated with protein synthesis [9]. NS1 can also directly modulate other important aspects of the virus replication cycle, including viral RNA replication, viral protein synthesis, and general host cell physiology, thereby enhancing virus pathogenicity [10–12]. Thus, NS1 protein has multiple functions associated with viral infection.

To gain further insight into the role of NS1 in regulating the host signaling cascade, as well as viral replication, we screened proteins from a human lung cell cDNA library using a T7-phage display system in order to identify proteins that interact with NS1(H5N1). Results of the screen identified a nucleolar and coiled-body phosphoprotein (Nopp140 or NOLC1) as an interacting protein. The interaction between these two proteins was further demonstrated by His-pull down experiments and Co-IP experiments *in vivo* [13]. NOLC1 is a 140 kDa nucleolar phosphoprotein which has been shown to exist in multiple forms with different molecular masses [14]. NOLC1 shuttles between the cytoplasm and the nucleolus, and interacts with many proteins [15], such as p80 coilin [16], NAP57 [17], protein kinase CK2 [18], etc. Binding of NOLC1 to snoRNPS can affect the modification of rRNA [19]. As a RNAP-interacting protein, NOLC1 can regulate the transcription of rRNA by binding to RNA polymerase I [20]. It is a component of the fundamental structure of the dense fibrillar and fibrillar center of nucleoli and has been implicated in the formation of the nucleolus during cell division [21]. All these function indicate that it can play an important role in cell proliferation, cell apoptosis, and tumorigenesis.

Transient expression studies revealed that NS1 protein is mainly localized in the nucleus, while NOLC1 protein is primarily localized to the nucleolus. Therefore, the two proteins share the same intracellular location, suggesting that their interaction is physiologically relevant [13]. Truncated effector domain studies of NS1 (H5N1) revealed that amino acids 104-200 are sufficient to allow for an interaction with NOLC1, and that 120-D and 195-R amino acids play the key role in the NS1-NOLC1 interaction [22]. Because the H5N1 viruses harbour a 5-codon deletion in their NS1, so 195R becomes 200R in the other IAVs. These two sites have been shown to be highly conserved in H5N1(A/Chicken/Hubei/327/2004 H5N1), H3N2(A/HongKong/489/97 H3N2), and H1N1(A/WSN/1940 H1N1). Therefore, we hypothesize that the NS1 protein of H3N2 and H1N1 can also interact with NOLC1 protein.

In order to validate our assumption, the interaction between NOLC1 and NS1 protein of H3N2 influenza virus was verified and demonstrated to promote apoptosis in A549 host cells, thus reducing virus replication. These findings provide new information on the biological function of the interaction between NS1 of the influenza virus and host protein NOLC1.

## RESULTS

### NS1 interacts with NOLC1

**Figure 1:**
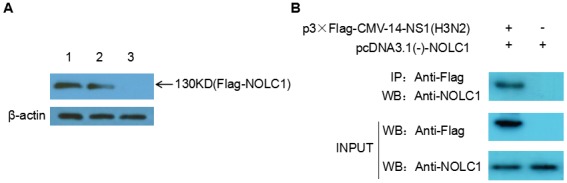
NS1 interacts with NOLC1 **(A)** NS1 and NOLC1 interaction determined by pull-down assay. Lane 1, Lysate from 293T cells expressing flag-NOLC1 was directly subjected to western blot analysis using anti-FLAG anitbody. Lane 2, Lysate from 293T cells expressing flag-NOLC1 that was incubated with His-tagged NS1-MagneHis™ beads and then eluted. Lane 3, Lysate from 293T cells expressing flag-NOLC1 that was incubated with MagneHis™ beads without adsorbed His-tagged NS1. **(B)** Interaction between NS1 and NOLC1 as determined by coimmunoprecipitation. Flag-NS1(H3N2) and NOLC1 were co-expressed in the 293T cells, the cell lysate was subjected to immunoprecipitation using anti-FLAG antibody, and the coimmunoprecipitated proteins were detected by western blot with the anti-NOLC1 antibody.

Our previous study indicated that 120-D and 195-R were the key sites for the interaction between NS1 of influenza virus H5N1 and host protein NOLC1. These sites are highly conserved in H5N1, H3N2, and H1N1, so we hypothesized that NS1 protein from H3N2 influenza virus could also interact with host protein NOLC1. A pull-down assay was performed in order to confirm this assumption. A His-NS1 fusion protein, expressed in E. coli BL21, was adsorbed on to MagneHis™ beads and then the beads were incubated with lysate obtained from 293T cells overexpressing a Flag-NOLC1 fusion protein. Proteins bound to the His-NS1 coated beads were eluted and analyzed by western blot using anti-FLAG antibody. As expected, a band at approximately 130 kD was detected. This band was not detected in the negative control, in which lysate of 293T cells overexpressing Flag-NOLC1 fusion protein was incubated with just MagneHis™ beads (Figure 1A).

Co-immunoprecipitation (Co-IP) was also performed to further confirm the interaction between NOLC1 and NS1. Expression vector p3×Flag-CMV-14-NS1(H3N2) and pcDNA3.1(-)-NOLC1 were co-transfected into the 293T cells, the cell lysate was subjected to immunoprecipitation using anti-FLAG antibody at 48h post-transfection, and the coimmunoprecipitated proteins were detected by western blot with the anti-NOLC1 antibody. The results showed that NOLC1 was associated with the NS1 protein(Figure 1B).

### NS1 reduces the quantity of NOLC1

**Figure 2:**
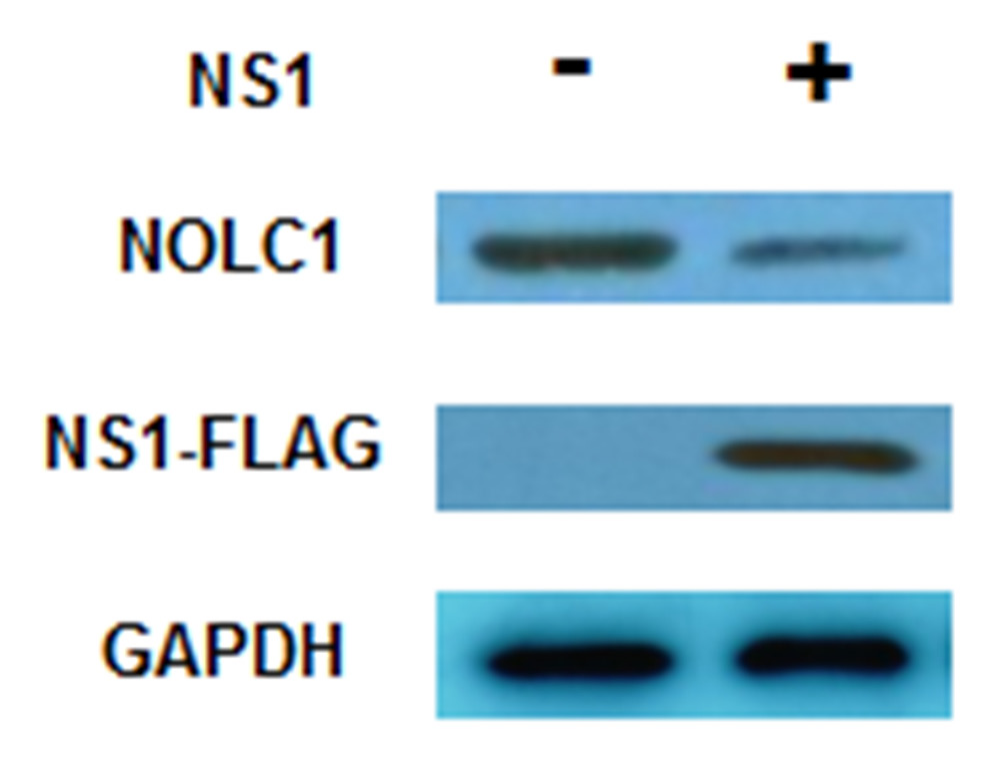
NS1 inhibits the synthesis of NOLC1 293T cells were cultured and transfected with p3×Flag-CMV-14-NS1(H3N2). The levels of NS1 and NOLC1 were detected at 48 h post-transfection by western blot analysis. GAPDH was used as an internal reference.

Previous studies have reported that NS1 protein can inhibit protein synthesis and increase virus protein replication [23–26]. An NS1 expression vector (p3×Flag-CMV-14-NS1) was constructed and transfected into 293T cells in order to determine whether or not the level of NOLC1 protein is affected by NS1 protein. The overexpression of NS1 protein in 293T cells resulted in a reduction in the quantity of NOLC1 observed on immunoblots, indicating that when NS1 is expressed, the synthesis of NOLC1 is reduced(Figure 2).

### Screening of the shRNA vector targeting NOLC1

**Figure 3:**
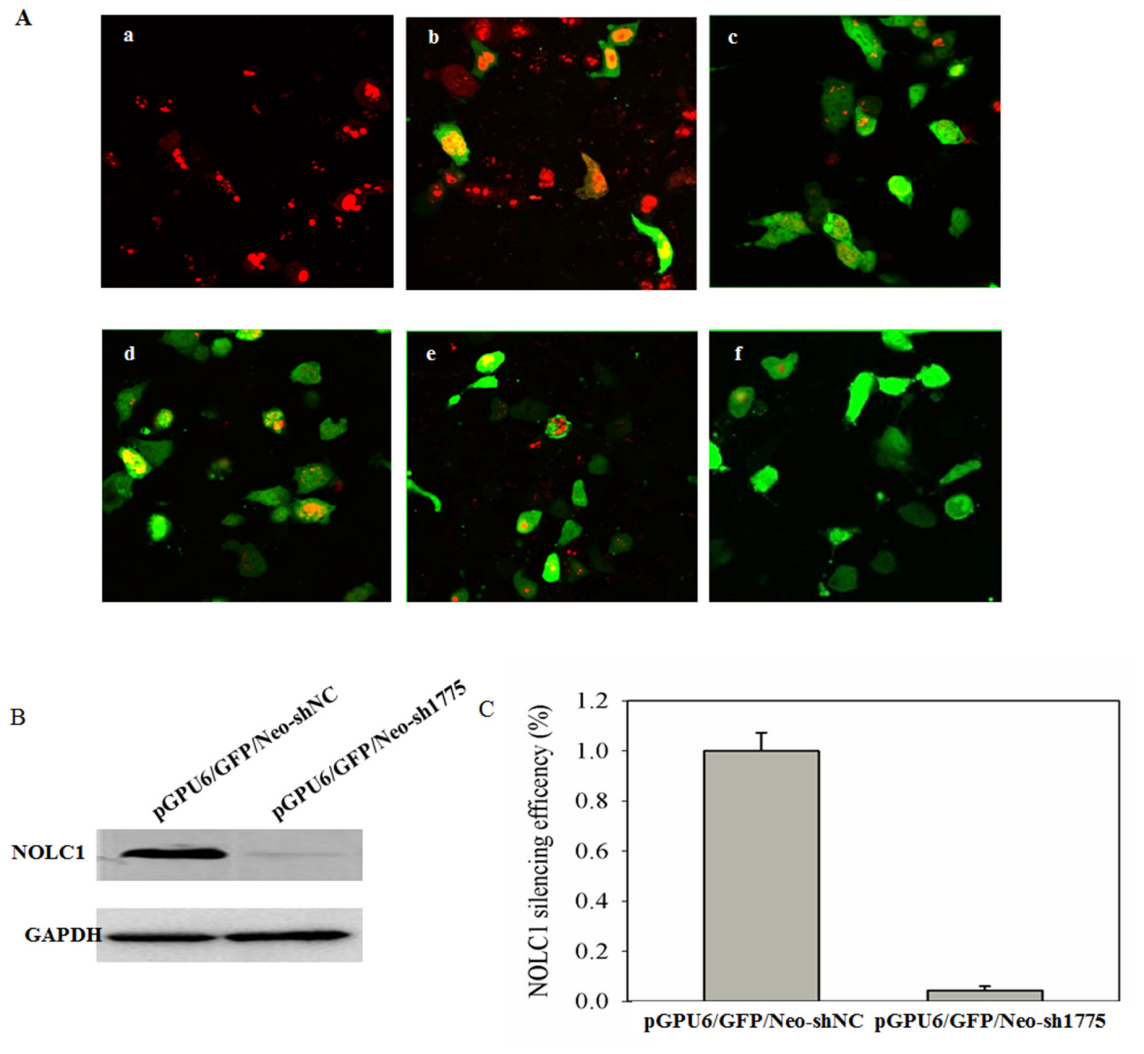
Screening of NOLC1-specific shRNA **(A)** Fluorescence detection of the RNAi. A549 cells were co-transfected with pDsRed-N1-NOLC1 and four different NOLC1 shRNA plasmids: pGPU6/GFP/Neo-sh667 (c); pGPU6/GFP/Neo-sh1262 (d); pGPU6/GFP/Neo-sh1376 (e); and pGPU6/GFP/Neo-sh1775 (f). After 48 h, each culture was observed and photographed with a confocal laser scanning microscope in order to compare the efficacy of the gene-silencing effect. A549 cells transfected with pDsRed-N1-NOLC1 was used as a blank (a) and A549 cells co-transfected with pGPU6/GFP/Neo-shNC and pEGFP-N1-NS1 were used as a negative control (b). **(B)** The effect of RNAi on NOLC1 expression as determined by western blot analysis. A549 cells were transfected with pGPU6/GFP/Neo-sh1775 plasmid and NOLC1 expression was analyzed by immunoblot analysis at 48 h post-transfection. A549 cells transfected with pGPU6/GFP/Neo-shNC plasmid was used as the negative control. **(C)** The staining intensity on the western blots were quantified using Totallab 2.0 software and statistically analyzed using SPSS V13.0. Values are the mean ± SD of three independent experiments performed in triplicate.

In order to select an effective NOLC1-specific shRNA vector, A549 cells were co-transfected with pDsRed-N1-NOLC1 and four different NOLC1 shRNA plasmids and then observed with a confocal laser scanning microscope at 48 h post-transfection, using the scrambled shRNA plasmid as a negative control. The silencing efficiency of shRNA was assessed by a decrease in DsRed expression. Fluorescence assays demonstrated that an obvious reduction in red fluorescence was induced by all four of the shRNA plasmids, especially by the pGPU6/GFP/Neo-sh1775 plasmid. In contrast, the negative control did not show any reduction in red fluorescence (Figure 3A). In order to further confirm the silencing efficacy of the shRNA plasmid, NOLC1 levels were measured by immunoblot analysis of A549 cells transfected with the pGPU6/GFP/Neo-sh1775 plasmid. The levels of immunostaining were determined at 48 h post-trasfection and quantified, as well as statistically analyzed, using Totallab 2.0 and SPSS V13.0 software, respectively. Results indicated that NOLC1 protein expression was significantly decreased relative to the negative control (P<0.01) (Figure 3B, 3C). Therefore, the pGPU6/GFP/Neo-sh1775 vector was selected to knock down the NOLC1 gene in the subsequent experiment.

### The interaction between NS1 and NOLC1 promotes apoptosis in A549 cells

**Figure 4:**
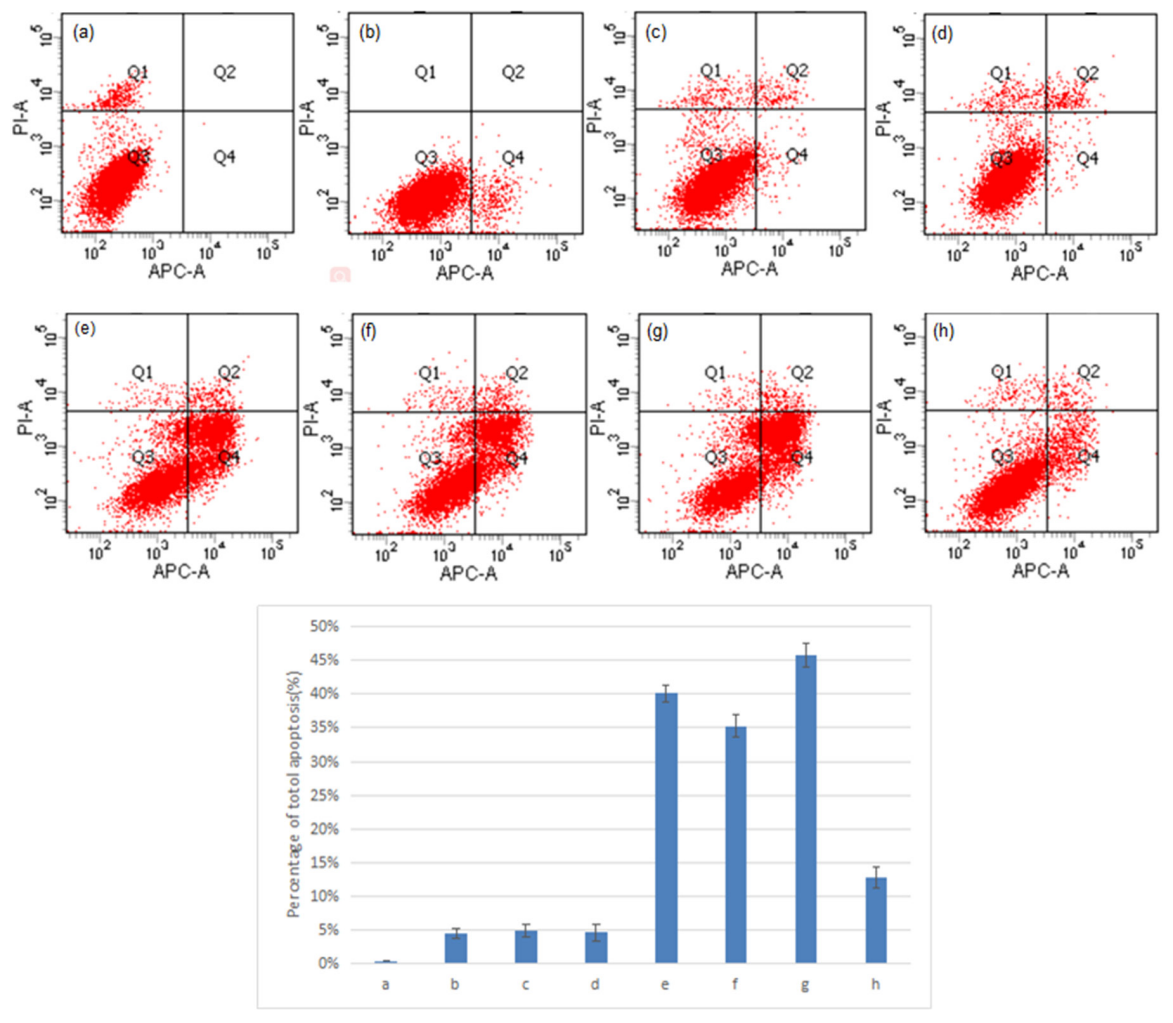
Double staining and flow cytometry analysis of cell apoptosis A549 cells transfected with different combinations of pGPU6/GFP/Neo-sh1775, pEGFP-N1-NS1, and pDsRed-N1-NOLC1 were double stained with Annexin V and PI, and the apoptosis index was determined by flow cytometry. Percent apoptosis for each of the sample-types are presented as the mean ± standard error from three separate experiments. The levels of apoptosis in transfected cell types in e – g were significantly different (p < 0.01) than the levels in the non-transfected and transfected cell types a – d. (P <0.01). (a) Cells without transfection; (b) cells transfected with pEGFP-N1; (c) cells transfected with pDsRed-N1; (d) cells transfected with pGPU6/GFP/Neo; (e) cells transfected with pEGFP-N1-NS1; (f) cells transfected with pGPU6/GFP/Neo-sh1775; (g) cells co-transfected with pEGFP-N1-NS1 and pGPU6/GFP/Neo-sh1775; (h) cells transfected with pEGFP-N1-NS1 and pDsRed-N1-NOLC1.

A549 cells were transfected with different combinations of pGPU6/GFP/Neo-sh1775, pEGFP-N1-NS1, and pDsRed-N1-NOLC1 in order to determine the effect of the interaction between NS1 and NOLC1 on host cell apoptosis. The level of apoptosis in the cells at 48 h post-transfection was assessed by double staining and flow cytometry. The degree of apoptosis in cells co-transfected with pDsRed-N1-NS1 and pGPU6/GFP/Neo-sh1775 was higher than cells transfected with pDsRed-N1-NS1 or pGPU6/GFP/Neo-sh1775 singly, while the the degree of apoptosis in cells co-transfected with pDsRed-N1-NS1 and pDsRed-N1-NOLC1 was lower. Cells not subjected to transfection and cells transfected with pEGFP-N1, pDsRed-N1, or pGPU6/GFP/Neo were used as negative controls and exhibited only minor levels of apoptosis (Figure 4).

### Suppression of NOLC1 protein reduces proliferation of the H3N2 influenza virus

In order to determine the effect of NOLC1 on the proliferation of H3N2 influenza virus, A549 cells were transfected with pGPU6/GFP/Neo-sh1775 to silence NOLC1 expression. The transfected cells were then infected with the H3N2 influenza virus. The virus titer (TCID50) was then examined at 48 h post-infection. Results indicated that the virus titer in cells where NOLC1 protein synthesis was suppressed was lower than in control cells (Table 1). These data indicate that the suppression of NOLC1 protein reduces the proliferation of H3N2 influenza virus in host cells.

**Table 1:** Suppression of NOLC1 protein synthesis reduces H3N2 influenza virus replication in A549 cells

Strand groups	n	Log TCID_50_^a^	P-value^b^	Fold reduction^c^
sh-NC	3	6.34±0.12	-	-
sh-NOLC1	3	5.06±0.09	<0.01	19

–, not applicable.

^a^The virus titer (TCID50) represents the mean value of three separate determinations in A549 cells.

^b^One-way ANOVA statistical analysis of log-transformed data, and mean comparison using a Dunnett’s method-test.

^c^Fold-reduction relative to the sh-NC treated group.

## DISCUSSION

Influenza A virus is highly infective, causing a large number of deaths in humans and many animal species each year [27]. NS1 protein is a non-structural protein encoded by the influenza A virus as a non-essential virulence factor. NS1, through its interaction with a variety of host proteins, has multiple functions, including changing the expression and redistribution of host proteins [28], and suppression of host immune responses [29, 30], thereby enhancing virus pathogenicity and virulence. Although NS1 protein has been reported to be involved in regulating apoptosis in infected cells, its role is contradictory, as NS1 has been reported to have both pro- and anti-apoptotic effects [31–33].

In our previous research, NS1 from H5N1 IAV was demonstrated to interact with NOLC1 protein in host cells. The 120-D and 195-R amino acids were the key sites determining the interaction, and these sites were highly conserved in H5N1, H3N2, and H1N1. Therefore, we hypothesized that the NS1 protein in H3N2 influenza virus could also interact with host protein NOLC1. This assumption was evaluated in the present study by examining the interaction between NOLC1 and the NS1 of H3N2 influenza virus using a pull-down and Co-IP assay. The results of the analysis confirmed our hypothesis. Based on the collective data we now predict that the interaction between NOLC1 and NS1 is universal and may play an important role in IAV infection.

NOLC1 is a nucleolar phosphoprotein associated with both the biogenesis of the nucleolus and the cell cycle [15], processes that play an important role in cell proliferation, cell apoptosis, and tumorigenesis. This further suggests that the interaction between NOLC1 and NS1 may play an important role in viral infection. We first tested the effect of NS1 on the expression of NOLC1 and found that NOLC1 levels decreased with an increase in NS1. Therefore, we envision that when NS1 is expressed during infection, it binds to a variety of host proteins and inhibits host protein synthesis, including NOLC1. At the same time, it also binds to NOLC1 protein and suppresses the normal function of NOCL1 in cells. Due to both properties, the quantity of functional NOLC1 is reduced. Next, we tested the effect of the NS1-NOLC1 interaction on cell apoptosis and found that overexpression of NS1 and silencing of NOLC1 can both induce apoptosis in A549 cells. Based on our data, it appears that NOLC1 protein plays a very important role in A549 cells, since down-regulation of NOLC1 induces apoptosis. We conclude that NS1 protein induces apoptosis in A549 cells by interacting with NOLC1 and by suppressing its synthesis. NS1 was overexpressed while NOLC1 was silenced in A549 cells in order to further validate this assumption. Results indicated that alterations in NS1 and NOLC1 levels induced higher levels of apoptosis. In contrast, when NOLC1 and NS1 were both overexpressed, apoptosis levels were lower. This suggests that the overexpression of NOLC1 compensated for the reduction in natural levels of NOLC1 resulting from the NS1-NOLC1 interaction, therefore, the level of apoptosis was not as great. Collectively, however, the data indicate that the interaction between NS1 and NOLC1 proteins induces apoptosis in A549 cells.

Apoptosis is a complex, fundamental biological process that eliminates extraneous cells during development, tumor development, normal homeostasis, and in response to disease. It was modulated by many biomolecules involving protein and ncRNAs [34, 35]. It also plays a critical role in defense against viral infection, although several viruses have evolved mechanisms to inhibit apoptosis. Host cells and the invading virus compete to control apoptosis [36, 37]. In our research, the interaction between NS1 and NOLC1 induced apoptosis in infected cells, which had a deleterious effect on the proliferation of the virus. This finding was supported by the virus titer analyses conducted in the current study. Virus titer was reduced 19 fold in infected A549 host cells in which NOLC1 was suppressed. This result indicates that NOLC1 protein plays an important role in virus proliferation. The collective data indicate that although NS1 protein plays an active role in IAV infection, the interaction between NS1 and NOLC1 has a negative impact on infection. These findings provide new information on the biological function of NS1 in IAV and provide a new perspective for understanding host antiviral defense mechanisms. In the next work, we will use computational models to predict and further study the correlation between the NOLC1 and the virus infection.

## MATERIALS AND METHODS

### Cell lines, vectors, virus and bacterial strains

H3N2 influenza virus(A/HongKong/489/97 H3N2) was obtained from a culture stored in the State Key Laboratory of virology of Wuhan University. A549, 293T cells, E. coli DH5a, and E.coli BL21competent cells, pET-28a, pEGFP-N1, pDsRed-N1, pCMV-flag, pcDNA3.1(-) and p3×Flag-CMV-14 plasmid, were all maintained in our laboratory. pGPU6/GFP/Neo-sh667, pGPU6/GFP/Neo-sh1262, pGPU6/GFP/Neo-sh1376, pGPU6/GFP/Neo-sh1775, pGPU6/GFP/Neo-shNC, and pGPU6/GFP/Neo-GAPDH were purchased from GenePharma (Shanghai, China).

### Reagents

DNA Marker DL2000, and restriction enzymes were purchased from TaKaRa (Dalian, China), Protein Marker was purchased from MBI (Lithuania), high purity plasmid (small) extraction kit was purchased from Beijing Union International Biological Gene Inc. (Beijing, China), IPTG was purchased from Beijing Bio-Tech Gene Technology LLC. (beijing, China), Ni-NTA His-bind Resins were purchased from Novagen (Germany)., Lipofectamine 2000 was purchased from Invitrogen (USA), Protease Inhibitor Cocktail was purchased from Merck (Germany). Protein A/G plus-Agarose beads were purchased from Promega (USA). anti-FLAG and anti-NOLC1 antibody was purchased from Santa Cruz (USA). Horseradish peroxidase labeled secondary antibody was purchased from Beijing Zhongshan Golden Bridge Company (Beijing, China). Western blot Luminol Reagent test kit was purchased from Santa Cruz.

### Cell culture and transfection

Cells were cultured in RPMI-1640 (Hyclone) with 10% (v/v) fetal bovine serum (FBS) (Solarbio), 100 U/ml penicillin and 100 μg/ml streptomycin (Solarbio), at 37°C in a 5% (v/v) CO_2_ humidified incubator. Cells were seeded into 6-well plates, grown to a density of 80% confluence and transiently transfected using Lipofectamine 2000 (Invitrogen) according to the manufacturer's protocol.

### Affinity-binding assays in the pull-down experiment

His-NS1(H3N2) fusion protein was expressed in E. coli BL21 and induced by IPTG. Five milligrams of His-NS1 was adsorbed onto MagneHis™ beads, as recommended by the manufacturer (Promega). The immobilized proteins were mixed with 200 μl of 293T cell lysate containing flag-tagged NOLC1 in a binding buffer (50 mM Tris-HCl, 150 mM NaCl, 1.0% Triton X-100, pH 7.6) containing a protease inhibitor mix (Roche) and incubated for 1 h at room temperature on a rotary shaker. Beads were precipitated and washed three times with 500 μl of binding buffer. Proteins bound to His-tagged-NS1 were analyzed by SDS-PAGE, followed by western blot analysis with anti-FLAG antibody.

### Affinity-binding assays in the Co-IP experiment

The recombinant plasmids p3×Flag-CMV-14-NS1(H3N2) and pcDNA3.1(-)-NOLC1 were constructed and co-transfected into 293T cells. After 48 h, the transfected cells were harvested and lysed at 4°C with RIPA lysis buffer. The homogenate was centrifuged and the supernatant was incubated with 5 μL of anti-FLAG polyclonal antibody and 20 μL protein A/G-plus beads for overnight at 4°C. The beads were collected and washed five times with PBS. The coimmunoprecipitated proteins were detected by western blot analysis with anti-NOLC1 antibody.

### Screening of shRNA targeting the NOLC1 gene

All of the oligonucleotide sequences used to produce NOLC1-specific shRNAs are shown in Table 2. Four shRNAs were designed based on the NOLC1 sequence (Homo sapiens strain, NCBI GenBank; Accession no. NC_004741.3) using Ambion's website tool to select potential shRNA (http://www.ambion.com/techlib/misc/siRNA_finder.html). The designed sense oligonucleotides contained a 4 nt overhang to create a BbsI restriction site (CACC) followed by a 21 nt sense siRNA sequence, a 9 nt loop (TTCAAGAGA), 21 nt reverse complementary antisense siRNA sequence, and a polymerase III terminator (TTTTTTG). The complementary antisense oligonucleotides contained a 4 nt overhang at 5′ terminal to create BamHI restriction sites (GATC). Two 62 nt-long annealed oligonucleotides with overhang ends were cloned into BbsI/BamHI restriction sites of the pGPU6/GFP/Neo (GenePharma) plasmid that contained the human U6 promoter. A non-silencing control (shNC) was similarly produced using a sequence that showed no significant identity to any human NOLC1 sequence data. The plasmid constructs were identified by BbsI and BamHI digestion and were subjected to nucleotide sequencing for confirmation. The resulting RNA transcripts were expected to fold back and form a stem-loop structure with a 21bp region homologous to the target sequences.

**Table 2:** Oligonucleotides used in shRNA construction

Name	Strand	Sequence
sh667	Top	5′-CACCGCCTGTCCAGAAGGGAGTTAATTCAAGAGATTAACTCCCTTCTGGACAGGCTTTTTTG-3′
	Bottom	5′-GATCCAAAAAAGCCTGTCCAGAAGGGAGTTAATCTCTTGAATTAACTCCCTTCTGGACAGGC-3′
sh1262	Top	5′-CACCGCAGTAGTCTCTAAAGCAACCTTCAAGAGAGGTTGCTTTAGAGACTACTGCTTTTTTG-3′
	Bottom	5′-GATCCAAAAAAGCAGTAGTCTCTAAAGCAACCTCTCTTGAAGGTTGCTTTAGAGACTACTGC-3′
sh1376	Top	5′-CACCGCTGGTACCACCAAGAATTCTTTCAAGAGAAGAATTCTTGGTGGTACCAGCTTTTTTG-3′
	Bottom	5′-GATCCAAAAAAGCTGGTACCACCAAGAATTCTTCTCTTGAAAGAATTCTTGGTGGTACCAGC-3′
sh1775	Top	5′-CACCGCTGAGAGCAGCAACAGTTCTTTCAAGAGAAGAACTGTTGCTGCTCTCAGCTTTTTTG-3′
	Bottom	5′-GATCCAAAAAAGCTGAGAGCAGCAACAGTTCTTCTCTTGAAAGAACTGTTGCTGCTCTCAGC-3′
shNC	Top	5′-CACC GTTCTCCGAACGTGTCACGT CAAGAGATTACGTGACACGTTCGGAGAATTTTTT G-3′
	Bottom	5′-GATCCAAAAAATTCTCCGAACGTGTCACGTAATCTCTTGACGTGACACGTTCGGAGAAC-3′

In order to select an effective NOLC1 shRNA vector, a NOLC1-DsRed fusion protein expression vector, pDsRed-N1-NOLC1 was constructed. A549 cells were co-transfected with pDsRed-N1-NOLC1 and four different NOLC1 shRNA plasmids; pGPU6/GFP/Neo-sh667, pGPU6/GFP/Neo-sh1262, pGPU6/GFP/Neo-sh1376, and pGPU6/GFP/Neo-sh1775. Transfected cells were observed with a confocal laser scanning microscope at 48 h post-transfection with the pGPU6/GFP/Neo-shNC vector serving as the negative control. The NOLC1 protein was fused with DsRed, so the silencing efficiency of RNAi could be assessed by a decrease in DsRed expression. In order to further confirm silencing efficacy, A549 cells were transfected with pGPU6/GFP/Neo-sh1775, and NOLC1 protein was detected by immunoblot analysis at 48 h post-transfection, using GAPDH as an internal control. Cells transfected with the pGPU6/GFP/Neo-shNC vector were used as a negative control. Western blots were scanned and densitometry analysis was performed using TotalLab 2.0 software (Nonlinear Dynamics, Durham, NC) according to the protocols provided by the vendor and the data was statistically analyzed using SPSS V13.0 software. All procedures were performed in triplicate and the data was expressed as a mean ± SD.

### Double staining and flow cytometry analysis of cell apoptosis

The NOLC1 expression vector, pDsRed-N1-NOLC1, and the NS1 expression vector, pEGFP-N1-NS1, were constructed. A549 cells were cultured in a 6-well plate (1×10^6^ cells/well) and transfected with different combinations of pGPU6/GFP/Neo-sh1775, pDsRed-N1-NOLC1, and pEGFP-N1-NS1. After 48 h, 1×10^6^ cells were collected and washed twice with ice-cold PBS, once with Binding Buffer (10 mM Hepes/NaOH, 140 mM NaCl, 2.5 mM CaCl_2_), and then resuspended in 200 μl of Binding Buffer. The cell suspension was then mixed with 5 μl fluorochrome-conjugated Annexin V for 15 mins, washed with Binding Buffer, and then mixed with 5 μl of Propidium Iodide staining solution in the dark for 15mins at room temperature (15-25°C). The percentage of apoptotic cells was determined by flow cytometry. A549 cells without transfection and cells transfected with pEGFP-N1, pDsRed-N1, or pGPU6/GFP/Neo were used as negative controls.

### Virus proliferation detection

To determine whether or not the silencing of NOLC1 reduced influenza virus production, the growing capacity of influenza A virus in A549 cells expressing siRNA was assessed. A549 cells were plated into six-well plates and cultured overnight at 37°C and 5% CO_2._ When cell layers reached 70-80% confluence, the A549 cells were transfected with 4ug of pGPU6/GFP/Neo-sh1775 and pGPU6/GFP/Neo-shNC. After 48 h, the transfected cells were infected with 1 MOI of H3N2 influenza virus, and supernatants were then harvested at 48 h post-infection from infected cells and virus titer (TCID_50_) in A549 cells was determined three times.

### Statistical analysis

All the experiments were performed using three independent trials run concurrently. Quantitative data are expressed as a mean ± SD. Statistical comparisons were evaluated using a One-way ANOVA. A probability value of P<0.01 was considered statistically significant.

## Abbreviations

NS1: non-structure 1
NOLC1: nucleolar and coiled-body phosphoprotein 1
PI: propidium Iodide
Co-IP: coimmunoprecipitation
